# Exploring the effect of the “quaternary regulation” theory of “peripheral nerve-angiogenesis-osteoclast-osteogenesis” on osteoporosis based on neuropeptides

**DOI:** 10.3389/fendo.2022.908043

**Published:** 2022-08-02

**Authors:** Shuhua Liu, Tongying Chen, Ruolin Wang, Hongxing Huang, Sai Fu, Yu Zhao, Shihao Wang, Lei Wan

**Affiliations:** ^1^ The Third Clinical Medical College of Guangzhou University of Chinese Medicine, Guangzhou University of Chinese Medicine, Guangzhou, China; ^2^ Department of Nephrology, Shenzhen Hospital (Futian) of Guangzhou University of Chinese Medicine, Shenzhen, China; ^3^ Department of Osteoporosis, The Third Affiliated Hospital of Guangzhou University of Chinese Medicine, Guangzhou, China

**Keywords:** neuropeptides, osteoporosis, bone metabolism, peripheral nerves, osteoporosis pain, quaternary regulation theory

## Abstract

Osteoporosis is a common bone metabolic disease among the middle-aged and elderly, with its high incidence rate and a major cause of disability and mortality. Early studies found that bone metabolic homeostasis is achieved through osteogenesis-osteoclast coupling. Although current anti-osteoporosis drugs can attenuate bone loss caused by aging, they present specific side effects. With the discovery of CD31^hi^ Emcn^hi^ blood vessels in 2014, the effect of H-type blood vessels on bone metabolism has been valued by researchers, and the ternary regulation theory of bone metabolism of “Angiogenesis-Osteoclast-Osteogenesis” has also been recognized. Nowadays, more studies have confirmed that peripheral nerves substantially impact bone metabolism. However, due to the complex function of peripheral nerves, the crosstalk mechanism of “Peripheral nerve-Angiogenesis-Osteoclast-Osteogenesis” has not yet been fully revealed. Neuropeptide serves as signaling molecules secreted by peripheral nerves that regulate blood vessels, osteoblasts, and osteoclasts’ functions. It is likely to be the breakthrough point of the quaternary regulation theory of “Peripheral nerve-Angiogenesis-Osteoclast-Osteogenesis”. Here, we discuss the effect of peripheral nerves on osteoporosis based on neuropeptides.

## Introduction

Osteoporosis (OP) is a common metabolic bone disease in the middle-aged and elderly population. It is mainly characterized by the aggravation of bone loss and the destruction of bone microstructure, leading to increased bone fragility and fracture risk (1). A multicenter epidemiological survey of OP in China in 2018 reported that the incidence rate of OP in men and women aged 50 and above was 6.46% and 29.13%, respectively. It is predicted that the population of osteoporosis will increase from 60 million to 120 million by 2050 (2). OP and osteoporotic fracture are also associated with high medical costs. It is estimated that the treatment cost of osteoporotic fracture in China will reach 19.92 billion US dollars by 2035 (3). The currently recognized pathogenesis of OP lies in the equilibrium between bone formation and bone resorption, and the primary treatment method is drug therapy, which requires long-term medication. At present, anti-OP drugs are mainly divided into bone resorption inhibitors and bone formation promoters. However, these medications are afflicted by poorly enduring side effects. For instance, Estrogen and calcitonin are not suitable for long-term use because of their carcinogenic risk, while bisphosphate and RANKL inhibitors may elicit atypical femoral fracture and mandibular osteonecrosis, and their incidence rate rises with time (4–8). Conversely, parathyroid hormone analog (PTHA) is a representative drug for promoting bone formation. However, it will raise the risk of osteosarcoma, and the drug withdrawal will provoke a vast bone loss, which alludes that excavating other factors that affect bone metabolism is imperative (4). Some studies have shown that atypical femoral fracture is a kind of stress fracture. The proximal and lateral femur is the tension side, and it bears large stress. Repeated stress stimulation will make periosteum hyperplasia and remodeling. Biphosphate and RANKL inhibitors will deposit here to inhibit bone remodeling, increase bone brittleness, and eventually lead to stress fracture (9). Biphosphate also has potential anti-angiogenic properties, which can inhibit the number of blood vessels in bone marrow, and may be one of the factors that increase the risk of bone necrosis in this part (10). Moreover, bone transplantation is an effective method for the treatment of traumatic bone defects. Studies reported that the vascularized and neuralized engineered bone implanted with sensory nerve and the vascular bundle has a higher degree of osteogenesis than simply vascularized bone fragments (11). Mounting studies have confirmed that peripheral nerve innervation is essential for normal bone metabolism. This means that we should not simply inhibit the activity of osteoclasts or promote the activity of osteoblasts in the treatment of osteoporosis. We should consider the role of blood vessels and peripheral nerves, that is, treating osteoporosis from the perspective of “quaternary regulation” may obtain greater benefits. Nevertheless, the mechanism of peripheral nerve regulation of bone metabolism has not been fully elucidated. Neuropeptides are important neurotransmitters of peripheral nerves. It is believed that in the near future, with the continuous deepening of research, the specific mechanism of peripheral nerve regulation of bone metabolism will be revealed. Here, we describe the effect of neuropeptides on bone metabolism in detail and help to enrich the quaternary regulation theory of “Peripheral nerve - Angiogenesis - Osteoclast - Osteogenesis” based on neuropeptides.

## Early bone metabolism regulation theory

The occurrence of osteoporosis is closely related to the disorder of bone metabolism. In initial studies, it was believed that the imbalance between osteoblast-mediated bone formation and osteoclast-mediated bone resorption was the cause of OP. The interaction of “osteogenesis and osteoclasts” is known as the “dual regulation theory” of bone metabolism, but this theory cannot fully explain the pathogenesis of OP. In March 2014, Kusumbe et al. found that the capillaries of the mouse skeletal system can be divided into H-type (CD31^hi^ Emcn^hi^) and L-type (CD31^Lo^ Emcn^Lo^). The abundance of H-type blood vessels can be used as a diagnostic index of vascular growth status and osteogenic ability, confirming the effect of H-type blood vessels on bone metabolism (12). In November 2014, Xie et al. found that osteoclast precursor cells can secrete platelet-derived growth factor BB (PDGF-BB), inducing H-type angiogenesis and promoting bone formation. Further animal experiments confirmed that PDGF-BB-deficient mice in the tartrate-resistant acid phosphatase-positive cell line showed a significantly lower bone mass and a reduction in the number of H-type blood vessels compared with wild-type, indicating that PDGF-BB played an essential role in coupling angiogenesis and bone formation (13). Therefore, the “ternary regulation theory” of bone metabolism was formally proposed, namely “Angiogenesis-Osteoclast-Osteogenesis”.

## Quaternary regulation theory of bone metabolism

The nervous system consists of the central nervous system and the peripheral nervous system, both of them have endocrine functions. The difference is that the central nervous system can release rich hormones (including calcitonin, parathyroid hormone, growth hormone, lean hormones, etc.) through neurohumoral regulation to affect bone metabolism (14). The effects of the central nervous system tend to be systemic, whereas the effects of the peripheral nervous system on bone metabolism tend to be local. Peripheral nerves are mainly involved in the regulation of the local bone microenvironment. However, the influence of peripheral nervous system on bone metabolism is very important. In 2005, Burt-Pichat B et al. (15) first confirmed that OVX-induced tibial bone loss in rats was related to decreasing nerve distribution density. In 2017, Grässel et al. (16) found that sensory and sympathetic neurotransmitters have trophic effects on bone and are related to the pathogenesis of OP. In 2018, Elefteriou et al. discussed in detail the effect of the autonomic nervous system on bone and found that sympathetic nerves (SNS) can negatively regulate bone mass, and parasympathetic nerves (PSNS) can regulate bone mass positively. The decrease of PSNS activity is related to osteoporosis (17). In 2019, Balasubramanian et al. (18) proposed that excessive activity of the autonomic nervous system is a sign of aging, providing new ideas for the treatment of OP. In December 2021, Xie et al. also found abundant sympathetic and parasympathetic fibers in bone tissue. The NE and Ach released from the nerve ending acted on the β2A receptor and M3 receptor on the surface of bone cells, respectively, stimulating and inhibiting the generation of neuropeptide Y (NPY) by bone cells. NPY can inhibit the cAMP/PKA/CREB signaling pathway and down-regulate the expression of the transcription factors Tead1 and Junb in bone marrow mesenchymal stem cells (BMSCs), resulting in the weakened osteogenic differentiation and enhanced adipogenic differentiation of BMSCs, which reveals a new mechanism mode of “nerve-bone axis” leading to osteoporosis (19). In January 2022, Mi et al. implanted electrodes into the dorsal root ganglia (DRG) at the L3 and L4 of the rat spine. They found that electrical stimulation of the DRG could activate the Ca^2+^/CaMKII/CREB signaling pathway and action potential, directly promoting the synthesis and release of CGRP, further inducing osteoporotic fracture healing and the formation of H-type blood vessels (20). Peripheral nerves can secrete neurotransmitters, neuropeptides, neurotrophic factors, among other signaling molecules, which can affect bone metabolism. In conclusion, neuropeptides can act as neurotransmitter signals and also act on adjacent tissues by paracrine regulation. Although XIE et al. (19) found the critical role of NPY in the bone microenvironment and revealed the regulatory pattern of the “nerve-bone axis”, the crosstalk mechanism of “Peripheral Nerve-Angiogenesis-Osteoclast-Osteogenesis” was not fully revealed. It is hoped that there will be more evidence to explain the “Quaternary Regulation” theory in the future.

## Peripheral nerves innervating bones and their characteristics

**Figure 1:**
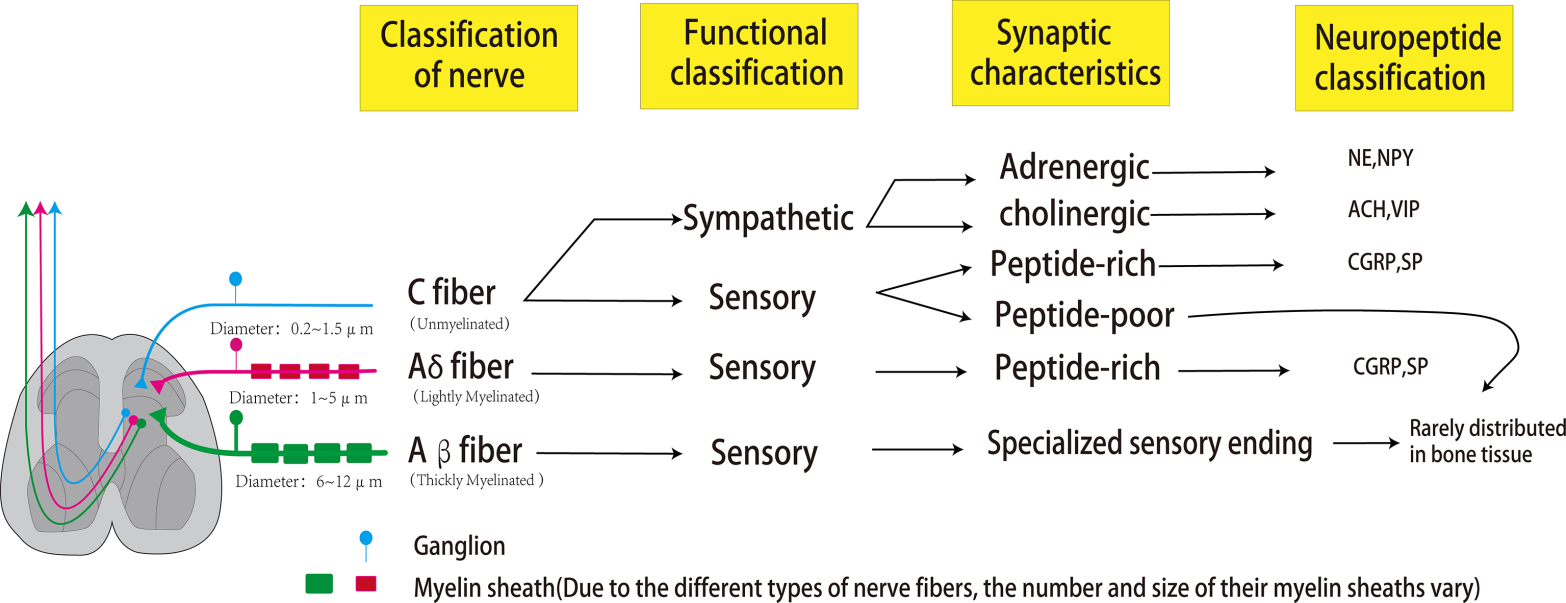
Peripheral nerves that dominate bones can be classified according to the size of axon diameter and the type of myelin sheath, which can be divided into C fibers, Aβ fibers, and Aδ fibers. The most abundant in bones is the unmyelinated C fiber with an axonal diameter of 0.2-0.5um and conduction velocity of 0.5-2m/s. Sympathetic nerves and some sensory nerves belong to the C-type nerve fibers. The sympathetic nerve has two phenotypes of adrenaline and cholinergic, which can release NPY (co-release with NE) and VIP (co-release with ACH), respectively. C-type sensory nerve fibers can be divided into peptide-poor and peptide-rich. Peptide-poor C-type fibers are rarely distributed in bones, while peptide-rich C-type sensory fibers can secrete CGRP and SP. The second common nerve fibers in bone are A-δ fibers, characterized by a thin myelin sheath, axon diameter of 1-5μm, and conduction velocity of 5-30 m/s. These fibers are peptide-rich fibers, which can also secrete CGRP and SP. Aβ fibers are characterized by a large axon diameter (6-12 um), thick myelin sheath, and fast conduction velocity (35-75 m/s). However, these fibers are rarely distributed in bones.

Bones are dynamic organs, and peripheral nerves are distributed in cortical and cancellous bone, periosteum, and bone marrow (21) (**Figure 1**). Sensory and autonomic nerve fibers are arranged in a fishnet-like arrangement on the periosteal surface to detect mechanical or chemical stimulation of cortical bone (21). The peripheral nervous system includes somatosensory nerves (sensory nerve fibers) and autonomic nerves (sympathetic and parasympathetic nerve fibers). Whether the autonomic nerves innervating the bone include parasympathetic nerves is still controversial. Some scholars believe that although the immunofluorescence of acetylcholine transporter (VACHT) and acetylcholine transferase (ChAT) exists in the bone microenvironment, some postganglionic sympathetic neurons also have the chemical characteristics of cholinergic nerves, and the postsynaptic neurons of the parasympathetic nerves are relatively short, whether they can directly reach and dominate the bone remains unclear (17, 22). Bajayo et al. (23) used the pseudorabies virus to trace the autonomic nerves innervating the skeleton and did not find that the posterior autonomic nerves have parasympathetic ganglia. However, it has been clear that the nerve fibers that dominate the bone include sensory nerve fibers, norepinephrine nerve fibers, and cholinergic nerve fibers.

The peripheral nerves dominating the bone can be classified according to the size of axons, the presence or absence of myelin sheaths, and the transmission speed. The most significant proportion of nerve fibers is C fibers with no myelin sheath, small axon diameter (0.2-0.5 μm in diameter), and slow transmission speed (0.5-2 m/s). C fibers can be divided into peptide-poor and peptide-rich fibers. Peptide-rich C fibers can secrete neuropeptides, such as calcitonin gene-related peptide (CRPG) and Substance P (SP). The second most common nerve fibers in bone are A-δ fibers with thin myelin sheath, moderate axon diameter (1-5µm in diameter), and moderate signal transmission speed (5-30 m/s). These fibers are peptide-rich fibers, which can also secrete CGRP and SP. Finally, A-β fibers with thick myelin sheath, large axon diameter (6-12μm in diameter), and fast conduction velocity (35-75 m/s) are relatively rare or absent in bone. Sympathetic nerve fibers dominating bones are C fibers with adrenergic or cholinergic phenotypes, the former secreting norepinephrine and neuropeptide Y (NPY), the latter secreting acetylcholine and vasoactive intestinal peptide (VIP) (24). A-β fibers usually detect non-noxious stimuli and can transmit subtle pressure changes (such as touch);in the same way, A-δ fibers are the primary fiber type involved in the transmission of pain stimuli and noxious mechanical stimuli (such as pressure and mechanical deformation); C fibers are mainly involved in the detection and transmission of noxious thermal, mechanical, and chemical stimuli (25). Functionally, since bones and joints are deeply located in the body, the lack of A-β nerve fibers may be related to the fact that bones and joints do not need fine touch sensation (26). The reason why unmyelinated and peptide-poor C fibers (abundantly expressed in the skin) are less abundant in bones and joints is not yet clear (26). XIE et al. (27) confirmed through animal experiments that the expressions of SP, CGRP and VIP in the tibia were significantly decreased in the OVX group, while NPY, NPY1R and NPY2R were significantly increased. Liu et al. (28) found that: OVX can lead to the decrease of TACR1, CGRP, CALCRL, NPY, and NPYY2 in the brain of mice, the increase of TACR1 in bone, and the decrease of SP, CALCRL, VIP, and VPAC2, confirming that the effect of estrogen deficiency on bone after ovariectomy is related to It is related to the regulation of SP, CGRP, VIP and NPY.

### CGRP

In 1982, CGRP was discovered in medullary thyroid cancer tissue. CGRP is a multifunctional neuropeptide consisting of 37 amino acids. After synthesis, CGRP will be transported to sensory nerve endings and stored in synaptic vesicles (29, 30). It was found that CGRP includes two subtypes (α and β). Mice lacking αCGRP showed decreased bone mass due to decreased bone formation rate, while mice lacking βCGPR showed an only mild and temporary reduction in bone formation, indicating that αCGRP can stimulate bone formation and βCGRP plays a minor role in osteogenesis (31, 32). CGRP receptor complex is composed of three independent proteins, including a G-protein coupled receptor (GPCR), receptor activity modifying protein 1 (RAMP1), and calcitonin receptor-like receptor (CLR). CGRP receptor is expressed in vascular endothelial cells, osteoclasts, osteoblasts, and bone marrow stromal cells (33–36).

#### CGRP inhibits osteoclasts activity

A growing number of studies have found that CGRP can inhibit the activity of osteoclasts and thus affect bone resorption. Although CGRP efficacy is lower than that of calcitonin, its role in inhibiting bone resorption has been valued by many researchers. It was found that after mouse bone marrow cells were induced and differentiated into osteoclasts by macrophage colony-stimulating factor (M-CSF) and nuclear factor-κB receptor activator of nuclear factor-κB ligand (RANKL). Only 0.1 nM concentration of CGRP can reduce the area of the absorption lacuna, and when the CGRP concentration needs to be greater than 10 nM, TRAP-positive cells can be formed, suggesting that the inhibitory effect of CGRP on osteoclast activity is more substantial than that on differentiation (33). In paraplegic patients, the bone mineral density in the paralyzed area decreases by 30-50% after one year, and the mechanism leading to increased bone resorption and rapid bone loss after paralysis is still unclear. Akopian et al. (37) found that the ability of progenitor cells in the bone marrow to form osteoclast-like cells increased by extracting bone marrow from paraplegic patients for *in vitro* culture and the formation of osteoclasts was significantly reduced after treatment with CGRP. Valentijn et al. (38) injected CGRP into OVX rats and found that it inhibited bone resorption-related indicators. It shows that CGRP can inhibit the activity of osteoclasts.

Monocytes/macrophages are the source of osteoclasts, meanwhile macrophages are considered to be an important cell population that regulates bone regeneration and osseointegration, and their polarized phenotype is particularly important. M1 macrophages are pro-inflammatory, while M2 Macrophages have anti-inflammatory properties. Studies have found that M1 macrophages may promote early and mid-stage osteogenesis, while M2 macrophages play an important role in matrix mineralization, and a proper switch from M1 to M2 phenotype is beneficial for fracture healing (39, 40). Yuan et al. constructed CGRP knockout mice and found that compared with the KO group, the CGRP^+/+^ group was more likely to induce more macrophages to transform to the M2 phenotype, which may be beneficial for peri-implant wound healing and osseointegration (41).

#### CGRP promote osteogenesis

Li et al. found the CGRP level significantly reduced in the bone marrow supernatant of elderly mice. *In vitro* experiments revealed that CGRP could promote osteogenic differentiation of BMSCs and inhibit adipogenic differentiation, indicating that CGRP may be a key regulator of age-related conversion between osteogenic differentiation and adipogenic differentiation of BMSCs be used to treat age-related bone loss (42). In the experiment of primary osteoblasts, CGRP promotes the proliferation and osteogenic activity of osteoblasts and their precursors in a dose-dependent manner by increasing the intracellular cAMP level and up-regulating the expression of activated transcription factors (34). XIANG et al. (43) constructed a CGRP gene knockout mouse model and found that after knocking out the CGRP gene, the amount of bone formation in mice was reduced. Targeting osteoblasts to express CGRP could increase the bone density of mice (31, 43, 44). Bone cement is a kind of medical material for orthopedic surgery. Studies have found that bone cement with CGRP can significantly enhance the proliferation of BMSCs, increase the activity of alkaline phosphatase in the process of BMSCs differentiation, and up-regulate the expression levels of osteogenic differentiation-related genes such as Bmp2, Osteonectin and Runx2 (45).

In conclusion, CGRP can inhibit bone resorption and promote bone formation *in vivo* and *in vitro*.

#### CGRP promotes angiogenesis

As one of the strongest vasodilators currently known, CGRP promotes bone formation partly because of its ability to dilate blood vessels and stimulate endothelial cell migration, promoting angiogenesis in bone remodeling (46). *In vitro* experiments have found that CGRP promotes endothelial cell proliferation and tube formation by enhancing the expression of vascular endothelial growth factor (VEGF), and this mechanism has been further verified in tumor tissues (47, 48). Bo et al. used CGRP to intervene in the co-culture system of human primary osteoblasts and human umbilical vein endothelial cells. CGRP can directly promote osteogenesis and indirectly promote osteogenesis by stimulating the differentiation of vascular endothelial cells. At the same time, CGRP can relax blood vessels, regulate the local blood flow of injury, accelerate blood supply, and jointly promote bone tissue repair (49). Distraction osteogenesis (DO), a surgical approach used to treat bone defects and limb lengthening, researchers injected CGRP into the area of ​​bone defects and found that CGRP enhanced vascularization and bone regeneration in a rat DO model (50). H-type blood vessels play an important role in coupling osteogenesis. Additionally, VEGF can promote the formation of H-type angiogenesis, indirectly indicating a positive correlation between CGRP and H-type angiogenesis.

### Substance P (SP)

SP is a peptide composed of 11 amino acids, widely distributed in the peripheral and central nervous systems, and belongs to the tachykinin family. SP receptors include NK-1, NK-2, and NK-3 receptors. Among them, SP has the highest affinity with the NK-1 receptor, which is the primary receptor of SP (51). NK1 receptors are not only found in cells of the nervous system and immune system, but also widely exist in osteoclasts, osteoblasts, osteocytes, epithelial cells, and vascular endothelial cells (52). SP is involved in many physiological and pathological processes, including angiogenesis and dilation, smooth muscle contraction, pain transmission, neurogenic inflammation, and bone metabolism (53). When SP receptor antagonist was used to block SP signal, the bone loss of OVX mice was aggravated, indicating that SP was of great significance to maintaining normal bone mass (54).

#### SP promotes bone resorption

SP was shown to induce osteoclastogenesis and enhance bone resorption activity by activating the transcription of NF-κB in bone marrow macrophages (55). Niedermair et al. (56) found the osteoclast apoptosis levels increased and a decreased rate of bone resorption after specific knockout of SP in a constructed kinin precursor 1 (Tac1)-deficient mice (Tac1 is the gene encoding SP). Hemokinin-1 (HK-1) ​​is also a member of the tachykinin family. HK-1 has a strong affinity for NK-1 receptors, but HK-1 does not affect the proliferation and differentiation of osteoclasts. Fukuda et al. divided bone marrow cells into two groups. The control group was intervened by 10^-7^M SP, and the experimental group was intervened by 10^-7^M SP and 10^−5^M HK-1. It was found that TRAP-positive multinucleated cells in the experimental group were significantly reduced, confirming that SP could promote osteoclast differentiation (57).

#### SP regulates osteogenesis

Both osteoblast precursor cells and bone marrow stromal cells express NK1 receptors. Wang et al. confirmed through *in vitro* cell experiments that SP could stimulate the proliferation and differentiation of bone marrow stromal cells in a dose-dependent manner. Low concentrations (10^-12^M) of SP can stimulate the expression of alkaline phosphatase, osteocalcin and Runx2 protein. A high SP concentration (10^-8^M) could enhance bone marrow stromal cells mineralization (55). Goto et al. used SP to intervene in rat osteoblasts and observed that SP could stimulate the expression of osteocalcin, Runx2, and type I collagen in the late osteogenic process. However, it could not produce the above effect in the early differentiation process, suggesting that SP could improve the osteogenic activity of late osteoblasts by acting on the NK1 receptor (58). Fu et al. (59) confirmed that SP could accelerate β-catenin translocation through the Wnt pathway to enhance osteogenic differentiation of BMSCs, and SP could also promote osteoblast differentiation and bone formation by increasing the production of cAMP and bone morphogenetic protein 2 (BMP-2). Fu et al. (60) used SP to intervene in BMSCs and found that SP promoted the expression of Bcl-2 and increased the ratio of Bcl-2 to Bax, confirming that SP inhibited the apoptosis of BMSCs through NK-1 receptors. Zhang et al. (61) found in the co-culture experiments of dorsal root ganglion (DRG) cells and BMSCs that DRG enhanced the autophagy level of BSMCs through the AMPK/mTOR signaling pathway, thereby maintaining the differentiation activity, and this process was related to the release of substance P. Therefore, SP can stimulate the proliferation, differentiation, and mineralization of pre-osteoblasts and improve the activity of late osteoblasts, but the specific mechanism needs to be further studied (62).

#### SP regulates angiogenesis

In a previous report, Liu et al. established a co-culture system of trigeminal ganglion sensory neurons and vascular endothelial cells. They found that when sensory neurons secreted SP increased, it could effectively promote the activation of vascular endothelial cells and promote angiogenesis, confirming that sensory neurons could directly promote angiogenesis through SP signal (63). Moreover, Kim et al. immobilized SP in nanofibrous materials to enable continuous release of SP and used this material to intervene in the hind limb ischemia model in mice. It was found that SP could promote the recruitment of mesenchymal stem cells into the ischemic area, promote angiogenesis, enhance tissue perfusion and prevent limb ischemic necrosis (64). Um et al. (65) reported that the number of circulating endothelial progenitor cells and CD31+ cells in peripheral blood increased after subcutaneous injection of SP, while CD31+ cells were associated with an angiogenic activity. In experiments in which sensory neurons (SNs) were co-cultured with endothelial cells, Leroux et al. found that CGRP and SP up-regulated angiogenic markers, including VEGF, angiopoietin 1, and Col4, and promoted angiogenesis (66). SP can promote angiogenesis, but whether it can target H-type blood vessels to affect bone metabolism still needs further research directions.

### NPY

NPY is a polypeptide composed of 36 amino acids and 1 carboxamide, expressed in the central nervous and peripheral nervous systems. NPY can be released into peripheral bone tissue through paracrine function. In sympathetic fibers of peripheral nerves, NPY is co-stored with norepinephrine, and when stimulated, both are co-released into bone tissue (67). Most mammals have the same NPY sequence, NPY is one of the known evolutionarily conserved peptides, and NPY plays a role by activating G protein-coupled receptors (Y receptors). At present, five known Y receptors are Y1, Y2, Y4, Y5, and Y6. NPY had the highest affinity with the Y2 receptor, followed by Y1 and Y5, and the lowest affinity with the Y4 receptor (67).

#### NPY acts on peripheral bone tissue through the Y1 receptor

Studies have found that the systemic knockout of Y1 and Y2 receptors can increase the cancellous bone mass, cortical bone mass, and osteoblast activity in mice, showing a protective effect on bone loss in OVX mice (68–70). The gene knockout of the Y4 receptor did not show changes in bone mass, osteoblast, and osteoclast activity, but there was a synergistic relationship between Y2 and Y4. The increased cancellous bone volume in mice with double gene knockout of Y2 and Y4 was more significant than in mice with a single gene knockout of Y2 (68). Y5 receptor is involved in angiogenesis, energy metabolism, and seizure control, while the Y6 receptor has no function in the human body (71). According to the review, the effect of NPY on bone metabolism mainly acts on the Y1 and Y2 receptors.

NPY can bind to specific receptors in target organs to regulate bone metabolism, which is considered a potential target for treating OP and promoting bone repair (51). The mechanism of NPY regulating bone metabolism is very complex, affecting the endocrine through the central nervous system, such as the expression of leptin, or directly affecting the peripheral Y1 receptor through the paracrine function peptidergic nerves (72). It was found that NPY regulated bone metabolism by directly acting on osteoblasts, and the expression of OPG was not related to Y2 receptor (73). Current research has found that Y1 and Y2 are expressed in the central nervous system, while osteoblasts express the Y1 receptor but not the Y2 receptor. The Y2 receptor in the central nervous system can regulate the function of the peripheral Y1 receptor (74, 75). This article mainly describes NPY and the role of Y1 receptors in the bone microenvironment.

When the Y1 receptor of mice was knocked out, bone formation and bone absorption increased simultaneously, but bone formation increased significantly (76). In this sense, bone marrow stromal cells extracted from Y1 receptor knockout mice were cultured under osteogenic conditions, and it was found that these bone marrow stromal cells could form more mineralized nodules, which not only increased the proliferation and differentiation ability of bone progenitor cells but also significantly enhanced the mineralization ability of mature osteoblasts (74). Khor et al. found that osteoclasts have Y1 receptors, and NPY can directly act on osteoclasts through Y1 receptors to inhibit bone resorption by regulating the expression of cAMP, RNKL, and OPG (73, 77). In summary, NPY directly inhibits the differentiation of mesenchymal stem cells and the activity of mature osteoblasts through the Y1 receptor, resulting in a high bone mass phenotype in Y1 receptor knockout mice. NPY can significantly inhibit bone formation and slightly inhibit bone resorption, but the overall phenotype is reduced bone mass and bone strength.

#### NPY can promote angiogenesis

In addition to its effects on osteoblasts and osteoclasts, NPY also regulates angiogenesis. Jiang et al. (78) intervened in vascular smooth muscle cells with NPY and found that NPY promoted the proliferation and migration of vascular epithelial cells and stimulated the growth of vascular smooth muscle. BIIE0246 is a Y2 receptor antagonist. Alasvand et al. (79) used this antagonist to intervene in tumor tissue and found that capillary density and VEGF level in tumor tissue decreased, confirming that NPY could regulate angiogenesis by activating the Y2 receptor on endothelial cells, but the specific mechanism was not precise. Other studies also confirmed that NPY at physiological concentrations promoted the synthesis of nitric oxide and VEGF release by activating Y2 and Y5 receptors, thereby inducing angiogenesis, and presented the therapeutic potential to promote the recovery of blood supply in ischemic tissues (80–82). Liu et al. found that NPY promoted osteoblast differentiation through the canonical Wnt pathway in a concentration-dependent manner at concentrations ranging from 10^-12^ to 10^-8^ mol/L, while that NPY treatment increased the migration of BMSCs and the expression of VEGF (83). This conclusion does not conflict with the above point, because NPY can exert different effects through different receptors.

### VIP

VIP was isolated from porcine intestines in 1970 and was named vasoactive intestinal peptide because of its vasodilating effect, which belongs to the glucagon/secretin superfamily. It was later found that VIP exists in the intestines and expresses in the central and peripheral nervous systems and can act as a neurotransmitter, so it was categorized as a neuropeptide. Furthermore, VIP is closely related to the occurrence of OP. In a cross-sectional study, Wang et al. (84) found that the expression level of VIP in serum of postmenopausal osteoporosis (PMOP) patients was lower than that of the healthy group, and the level of VIP was positively correlated with the bone mineral density of lumbar 1-4 and total hip.

VIP is abundant in the periosteum, and VIP nerve fibers are mainly distributed in the Haversian and Volkmann’s canals. As early as 1986, Hohmann et al. (85)found that the nerve fibers that dominate the bone and periosteum can regulate bone mineralization by expressing VIP. After the resection of the sympathetic postganglionic fibers, the VIP release was inhibited, and further studies found that VIP and acetylcholine coexist in vesicles, confirming that the sympathetic nerve releases VIP with the cholinergic phenotype. VIP receptors include the VPAC1 receptor, VPAC2 receptor, and PAC1 receptor. Moreover, the VPAC1 receptor and VPAC2 receptor have similar affinity to VIP and Pituitary adenyl cyclase active peptide (PACAP), while the PAC1 receptor has 100-1000 times higher affinity to PACAP than VIP (86).

#### Effects of VIP on bone metabolism and blood vessels

Both osteoblasts and osteoclasts have functional receptors for VIP, and VIP regulates the bone formation, metabolism, and remodeling by specifically binding to receptors on different cells (87). In this sense, Lundberg et al. (88) proved by RT-PCR that unmineralized mouse skull osteoblasts expressed VPAC2 but did not express VPAC1 or PAC1. However, after induced osteoblast mineralization, it can express VPAC1. Furthermore, Ransjö *et al.* (89) demonstrated that mouse bone marrow osteoclasts expressed VPAC1 and PAC1 receptor mRNA but not VPAC2 mRNA.

Observing the morphology of osteoclasts in rat bone marrow, researchers found that VIP can lead to cytoplasmic contraction of osteoclasts and decreased osteoclast motility (89). The binding of VIP to the VPAC1 receptor can inhibit the aggregation of osteoclasts and weaken the bone resorption activity of osteoclasts. Additionally, VIP can also stimulate osteoblasts and downregulate osteoclastogenesis by promoting cAMP production (90). VIP binds to VPAC2 receptors in osteoblasts, activates AMP and ERK signaling pathways, and increases the ratio of RANKL/OPG, confirming the important role of VIP in bone remodeling (91)Liu et al. (92) confirmed that VIP could promote osteogenic differentiation of rat bone marrow mesenchymal stem cells *in vitro* by activating Wnt/β-catenin signaling pathway. Besides, the team used the sympathetic resection mouse model constructed by 6-hydroxydopamine (6-OHDA). Based on the model, the femur fracture model was established. It was found that the VIP expression in the fracture site of the sympathetic resection mouse was significantly reduced, and the expression levels of osteocalcin (OCN) and osteopontin (OPN) were reduced. VIP treatment could inhibit bone resorption and rescue the inhibitory effect of 6-OHDA on bone remodeling (93).

Although many studies have confirmed that VIP can inhibit osteoclast activity, the effects of VIP on osteoblasts and osteoclasts are not mutually exclusive. VIP binds to the VPAC2 receptor of osteoblasts to activate protein kinase A, thereby stimulating the production of IL-6, which can promote osteoclast activity (94). In the case of co-culture of osteoclasts and osteoblasts, after adding VIP, osteoclasts will be inhibited in the early stage, but with the extension of VIP stimulation, osteoclasts will get rid of the initial inhibition and gradually recover their activity. It indicates that VIP has a dual regulatory function on osteoclast activity (95). The existence of dual regulation may be that the VIP receptors of osteoblasts and osteoclasts are different, and the affinity of each receptor to VIP is different, resulting in different effects of VIP on osteoclasts at different times and concentrations. The mechanism of VIP on osteogenesis and osteoclast still needs further research.

Furthermore, VIP is an endogenous vasodilator. Intravenous injection of VIP can lead to strong vasodilation and reduced blood pressure. VIP and its receptors are potential targets for migraine drugs (96). *In vitro* experiments revealed that VIP can also stimulate tube formation in human umbilical vein cells and increase VEGF expression during the osteogenic differentiation of rat bone marrow mesenchymal stem cells (93). The concentration of serum VIP correlated with the severity of non-traumatic femoral head necrosis, which may be related to the role of VIP in promoting angiogenesis (97).

## Discussion

There is an interaction between peripheral nerve and bone tissue. Bone cells can secrete BMPs, chemokines, axon guidance factors, neurotrophic factors, and CGRP to regulate the development and repair of the nervous system, indicating that there is a crosstalk between bone cells and peripheral nerve, which is essential for regulating the dynamic balance of bone growth, and repair.

Correct innervation is necessary for fracture repair and bone development. Neurological changes and abnormal levels of specific neuropeptides are thought to be partly responsible for degenerative changes in the bones, such as OP and osteoarthritis. Although neuropeptides and their receptors are not the only factors in the regulation of peripheral nerves on bone, neuropeptides are one of the mediators, but their role should be monitored. At present, neuropeptides are considered one of the targets for the treatment of OP, but the clinical evidence of the application of neuropeptides and their receptor antagonists or agonists in OP still has significant limitations. Although neuropeptides have essential effects on bone metabolism, it is still a long way to treat them as drugs for OP.

**Figure 2:**
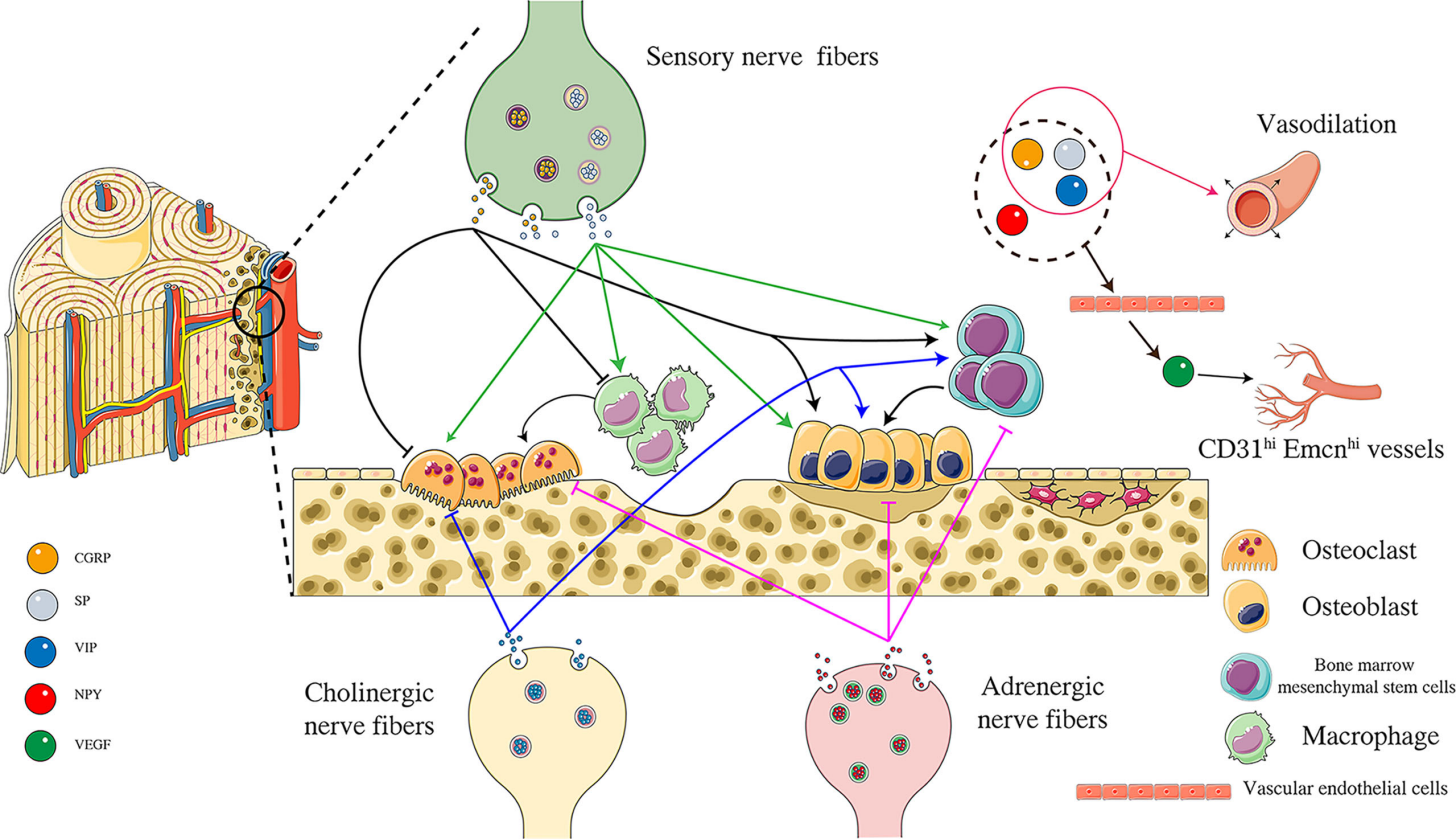
Effects of different neuropeptides on “Angiogenesis-Osteoclast-Osteogenesis”. CGRP released from peripheral sensory nerve endings can inhibit the activity of osteoclasts and inhibit the differentiation of macrophages to osteoclasts by acting on CGRP receptors. At the same time, CGRP can promote the activity of osteoblasts and the osteogenic differentiation of bone marrow mesenchymal stem cells. SP is another neuropeptide released from sensory nerve endings, promoting the differentiation of macrophages into osteoclasts and improving osteoclast activity by acting on NK1 receptors. SP can also promote osteoblast activity and bone marrow mesenchymal osteogenic differentiation of stem cells. NPY is released from adrenergic nerve endings. NPY is released from adrenergic nerve endings, and NPY acts on peripheral Y1 receptors to inhibit osteoblast activity, osteogenic differentiation of bone marrow mesenchymal stem cells, and osteoclast activity. VIP is released from acetylcholinergic nerve endings, inhibits osteoclast activity by binding to VPAC1 receptor, and can also promote osteoblast activity by binding to VPAC2 receptor. VIP also promotes osteogenic differentiation of bone marrow mesenchymal stem cells. CGRP, SP, NPY, VIP can promote VEGF production indirectly promote the formation of CD31^hi^ Emcn^hi^ blood vessels. In addition, CGRP, SP, and VIP have the effect of vasodilation. Figure 2 was modified from Servier Medical Art(http://smart.servier.com/), licensed under a Creative Common Attribution 3.0 Generic License. (https://creativecommons.org/licenses/by/3.0/).

Neuropeptides are a complex system. Neuropeptides have opposite effects on bone cells due to different concentrations and receptor-ligand interactions (**Figure 2**). Furthermore, neuropeptides do not act independently, and the relationship between neuropeptides is also worthy of further exploration. It was found that both SP and CGRP could promote the expression of BMP2 and Runx2 and induce mineralization in mouse osteoblasts *in vitro*. However, when SP and CGRP were used in combination, the BMP2 signal was down-regulated and osteogenic differentiation was inhibited, suggesting that there might be an interaction between these two neuropeptides (98). Among the four neuropeptides described in this paper, CGRP is the most studied, showing prominent promotion of bone formation and angiogenesis in various aspects. Experimental results show that magnesium-containing intramedullary nails can stimulate local CGRP release, promote osteogenic differentiation of periosteal stem cells, and accelerate femoral shaft fracture healing in OVX mice (99). Whether similar mechanisms can be used to treat osteoporotic fractures is worthy of further study. All four neuropeptides have been confirmed to promote VEGF expression, thereby promoting angiogenesis. Although it has not been reported that these four neuropeptides can directly target H-type vascular endothelial cells, VEGF is an important promoter of H-type vascular differentiation. Neuropeptides have regulatory effects on nerves, osteoblasts, osteoclasts, and blood vessels and are likely to be an essential factor in the crosstalk of “Peripheral Nerve-Angiogenesis-Osteoclast-Osteogenesis”. Neuropeptide is an important entry point to improve the theory of “quaternary regulation theory”.

The peripheral nerves that innervate the bones include sensory nerves. When talking about sensory nerves, we must mention the transmission of pain signals by sensory nerve fibers. Pain is one of the most terrible complications of OP, and about 85% of OP patients suffer from bone pain (100). Pain is also the most direct and typical symptom of OP and is one of the main reasons OP patients seek medical attention. Both OP and osteoporotic fractures are related to chronic bone pain. At present, the mechanism of chronic pain in OP is still unclear. Nonsteroidal anti-inflammatory drugs or opioids are generally used according to the degree of pain, but long-term use of opioids will reduce bone mineral density, cause gastrointestinal damage, renal toxicity, and addiction (101). Moreover, studies have confirmed that CGRP, SP, and VIP can induce peripheral pain maintain microglia activation and central sensitization (102, 103). However, NPY can mediate analgesic effects through Y1 and Y2 receptors, and intrathecal injection of NPY can relieve neuropathic, inflammatory pain, or postoperative pain (104). The density of bone innervation is related to the severity of bone pain after an injury. Reducing the density of CGRP-positive nerve fibers can effectively reduce the pain caused by fractures (105). Promoting bone metabolism and inhibiting pain are the best strategies for treating OP. Neuropeptides are a new way to achieve this therapeutic strategy.

## Author contributions

SL conceived, wrote, and revised this manuscript. TC collected materials and revised the manuscript. RW made the first revision of the manuscript. SF and YZ contributed to manuscript drafting. SW submitted the final manuscript. LW and HH made the final manuscript revisions. All authors contributed to the article and had final approval of the submitted and published versions.

## Funding

This work was supported by the National Natural Science Foundation of China (Grant No.81973886 and 82174395), by Guangzhou University of Chinese Medicine “Double First-Cass” and High-Level University Discipline Collaborative Innovation Team Project (Grant No. 2021XK21), by Natural Science Foundation of Guangdong Province(Grant No. 2022A1515012067), and by Guangdong Graduate Education Innovation Project (Grant No. 2022XSLT013).

## Conflict of interest

The authors declare that the research was conducted in the absence of any commercial or financial relationships that could be construed as a potential conflict of interest.

## Publisher’s note

All claims expressed in this article are solely those of the authors and do not necessarily represent those of their affiliated organizations, or those of the publisher, the editors and the reviewers. Any product that may be evaluated in this article, or claim that may be made by its manufacturer, is not guaranteed or endorsed by the publisher.
